# Application and Comparison of the MODIS-Derived Enhanced Vegetation Index to VIIRS, Landsat 5 TM and Landsat 8 OLI Platforms: A Case Study in the Arid Colorado River Delta, Mexico

**DOI:** 10.3390/s18051546

**Published:** 2018-05-13

**Authors:** Christopher J. Jarchow, Kamel Didan, Armando Barreto-Muñoz, Pamela L. Nagler, Edward P. Glenn

**Affiliations:** 1 Soil, Water, and Environmental Science (SWES), University of Arizona, Tucson, AZ 85719, USA; 2 US Geological Survey, Southwest Biological Science Center, University of Arizona, 520 N. Park Avenue, Tucson, AZ 85719, USA; pnagler@usgs.gov; 3 Biosystems Engineering, University of Arizona, Tucson, AZ 85719, USA; didan@email.arizona.edu (K.D.); abarreto@email.arizona.edu (A.B.-M.); 4 Environmental Research Laboratory, University of Arizona, Tucson, AZ 85719, USA; EGlenn@ag.arizona.edu

**Keywords:** remote sensing, EVI, Landsat, MODIS, VIIRS, Colorado River Delta

## Abstract

The Enhanced Vegetation Index (EVI) is a key Earth science parameter used to assess vegetation, originally developed and calibrated for the Moderate Resolution Imaging Spectroradiometer (MODIS) aboard the Terra and Aqua satellites. With the impending decommissioning of the MODIS sensors by the year 2020/2022, alternative platforms will need to be used to estimate EVI. We compared Landsat 5 (2000–2011), 8 (2013–2016) and the Visible Infrared Imaging Radiometer Suite (VIIRS; 2013–2016) to MODIS EVI (2000–2016) over a 420,083-ha area of the arid lower Colorado River Delta in Mexico. Over large areas with mixed land cover or agricultural fields, we found high correspondence between Landsat and MODIS EVI (R^2^ = 0.93 for the entire area studied and 0.97 for agricultural fields), but the relationship was weak over bare soil (R^2^ = 0.27) and riparian vegetation (R^2^ = 0.48). The correlation between MODIS and Landsat EVI was higher over large, homogeneous areas and was generally lower in narrow riparian areas. VIIRS and MODIS EVI were highly similar (R^2^ = 0.99 for the entire area studied) and did not show the same decrease in performance in smaller, narrower regions as Landsat. Landsat and VIIRS provide EVI estimates of similar quality and characteristics to MODIS, but scale, seasonality and land cover type(s) should be considered before implementing Landsat EVI in a particular area.

## 1. Introduction

Vegetation Indices (VIs) calculated from airborne or satellite imagery have been used to assess a multitude of plant characteristics. For example, the widely-used Normalized Difference Vegetation Index (NDVI) captures and responds to actively-photosynthesizing plant tissues [1,2] and has been used extensively as a proxy for traits like plant biomass, leaf area index and canopy cover (e.g., [1,3,4,5,6,7,8]). NDVI is a key Earth Science Data Record (ESDR); however, issues with saturation over dense canopies and the impact of below canopy soil color remain a serious challenge [4]. Other spectral indices have been proposed for a more accurate evaluation of specific biophysical parameters, such as the Soil Adjusted Vegetation Index (SAVI; [9]) and the Modified Soil Adjusted Vegetation Index (MSAVI; [10]). These indices leverage differences in the reflectance of materials in the red and near-infrared (NIR) regions of the spectrum, thereby allowing for the identification or quantification of target materials in the landscape.

In addition to the red and NIR, other VIs include the blue band and correction coefficients that adjust reflectance values to control for factors such as background scattering and atmospheric resistance. One such index that is the focus of the current study is the Enhanced Vegetation Index (EVI), calculated as:

(1) $$\mathrm{EVI}=G\frac{\left(\rho \mathrm{NIR}-\rho \mathrm{red}\right)}{\left(\rho \mathrm{NIR}+C_{1}\times \rho \mathrm{red}-C_{2}\times \rho \mathrm{blue}+L\right)},$$

where G is a gain factor (set at 2.5), *C*_1_ and *C*_2_ correct for aerosol resistance (set at 6 and 7.5, respectively), L adjusts for canopy background (set at one) and *ρ*_NIR_, _red_ and _blue_ are reflectances in the near infrared, red and blue wavelengths, respectively [4,11,12]. The standard Moderate Resolution Imaging Spectroradiometer (MODIS) EVI product is available as radiometrically-calibrated and atmospherically-corrected 16-day composites, where the highest quality pixels over a given 16-day period are selected to represent the period [4,12]. The gain factor (G), *C*_1_, *C*_2_ and L coefficients were all derived and optimized for the MODIS sensor on the Terra and Aqua Satellites [4,11]. These coefficients improve the vegetation signal by suppressing canopy background and correcting for aerosols or residual aerosols (in the case of full atmospheric correction; [4,13]), allowing the index to respond more to characteristics like the Leaf Area Index (LAI) and canopy structure compared to the widely-used NDVI [14]. As a result, EVI has been used as a robust estimator of biophysical parameters like evapotranspiration [15,16,17].

Launched aboard Terra and Aqua satellites on 18 December 1999 and 4 May 2002, respectively, MODIS sensors have exceeded their design lifetime of six years and continue to capture images every 1–2 days, forming a long-term record of NDVI and EVI from 2000 to the present [18,19,20]. Because of this and the high quality of MODIS VI products [21], these data continue to be important components of remote sensing research and a critical ESDR. Using the phrase “MODIS EVI”, a search of scholarly articles in Google Scholar returned 21,800 results, with 9990 published since 2010. Despite heavy use, the National Aeronautics and Space Administration plans to decommission the satellites by 2020/2022, without a direct substitute [22]. The Visible Infrared Imaging Radiometer Suite (VIIRS) aboard the Suomi National Polar-orbiting Partnership spacecraft launched in December 2011 is slated to replace MODIS and will provide Earth system data for the next 30 years [23].

VIIRS will continue the MODIS tradition of providing standard, well-characterized and consistent land surface observations [24]. Therefore, it is critical to understand the performance of EVI from alternative sensors, especially as inputs to empirically-developed and MODIS-derived ecohydrological algorithms (e.g., [25]). VIIRS captures daily images at a nadir resolution of 375 m in the NIR and red bands and 750 m in the blue and shortwave infra-red bands. VIIRS EVI is calculated using the same equation as MODIS EVI after an initial implementation with a G factor of two compared to 2.5 for MODIS (see Equation (1); [24]). The standard MODIS equivalent VI product suite will follow the exact same methodology of the Earth Observing System-MODIS, producing NDVI, EVI and EVI2 [26], with plans to replace EVI with the two-band EVI2, which shows superior performance over areas with sub-pixel clouds and aerosols [27]. EVI2 and EVI are functionally the same, without the need for the blue band [28].

More recently, the U.S. Geological Survey’s (USGS) Earth Resources Observation and Science (EROS) Center has applied the MODIS-based EVI algorithm (with MODIS-optimized coefficients) to imagery acquired by the Landsat 4–5 Thematic Mapper (TM), Landsat 7 Enhanced Thematic Mapper Plus (ETM+) and Landsat 8 Operational Land Imager (OLI) satellite platforms [29]. The Landsat archive is the longest running civilian satellite series in the world, with data available from 1972 to the present [30]. The standard EVI product from the USGS is generated from atmospherically-corrected (surface reflectance) data and can be obtained for periods ranging from 1982 (Landsat 4 TM) to the present (Landsat 7 ETM+ or Landsat 8 OLI), with a 16-day repeat cycle. Though both Landsat 7 ETM+ and Landsat 8 OLI are presently capturing images, a failure of the Landsat 7′s Scan Line Corrector on 31 May 2003 resulted in streaks of missing data, limiting the utility of these images. One potential advantage to applying the EVI algorithm to Landsat-based data over MODIS for small study sites is a higher spatial resolution (30 m for TM, ETM+ and OLI) that MODIS and VIIRS cannot attain (e.g., [16]). However, to our knowledge, the performance of the standard Landsat EVI product has not been formally compared to MODIS EVI. This represents an opportunity and important area of research, as both the resolution and impending decommissioning of MODIS will necessitate the use of alternative platforms for deriving EVI in the future.

Our objective was to assess the relationship between MODIS, Landsat and VIIRS-derived EVI data for corresponding time periods and geographic locations in support of multi-sensor continuity and remote sensing-based biophysical parameter validation. We acquired and compared temporally- and geographically-coincident early, mid and late growing season EVI scenes of all four platforms from 2001–2016 for the lower Colorado River Delta (CRD) in Mexico, an area totaling about 420,083 ha. Although MODIS and VIIRS are typically applied to significantly larger areas, we purposefully chose a smaller area for our comparison, as the application of MODIS data to such scales has increased in recent years (e.g., [16,31,32,33]) and this allowed us to more easily control the effect of extraneous variables. The CRD is also well characterized by a variety of land cover conditions (i.e., soil and vegetation), allowing for a comparison across different surface types, vegetation conditions and scales. We further sub-divided our analysis by sensor, size (area) and land cover type and provided band-specific comparisons to help explain differences between these key sensors and their respective data records.

## 2. Materials and Methods

### 2.1. Study Region

**Figure 1:**
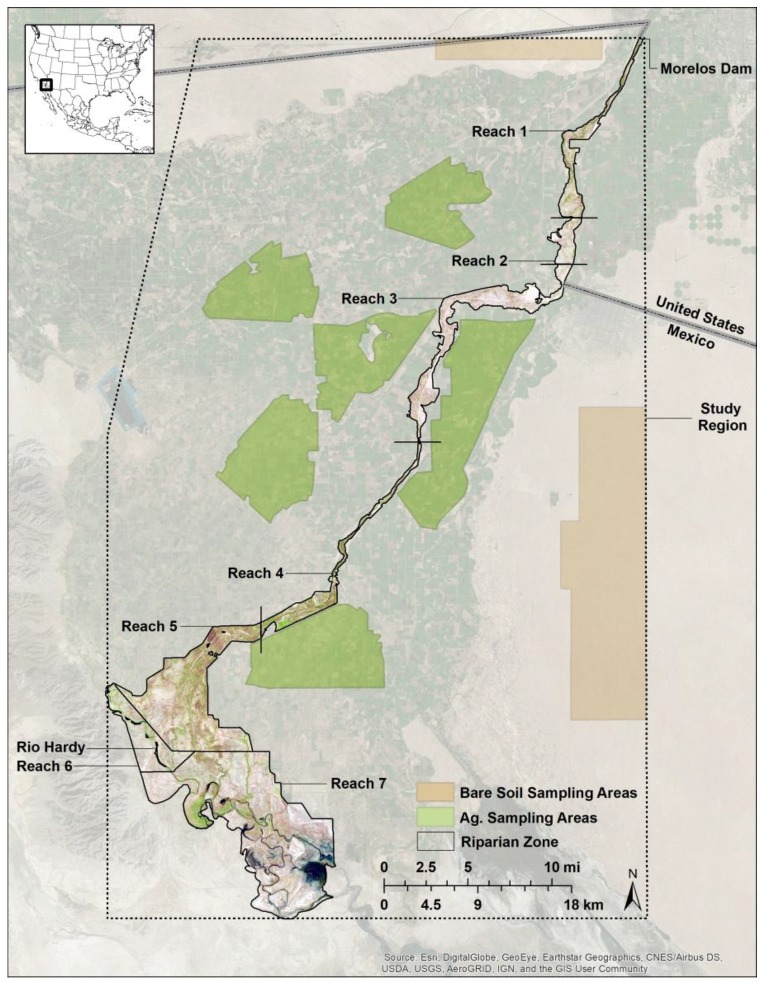
The riparian zone (separated by reach), bare soil and agricultural (ag.) sampling areas and the study region analyzed.

We studied the portion of the CRD’s riparian zone (corridor; 30,695 ha) and surrounding area (the study region; 420,083 ha) extending from the U.S.-Mexico border to the intertidal zone of the Sea of Cortez in Mexico (Figure 1). This portion of the Colorado River has been heavily modified by human activities, with extensive agricultural fields and flood-control levees forming the majority of the eastern and western boundaries of the riparian zone. Although this region is arid (averaging ~63 mm precipitation annually), it historically received more than 15 billion cubic meters of annual surface flows, primarily from snow melt in the upper Colorado river basin (summarized in [34]). Because of upstream diversions and impoundments, the CRD is now mostly dry, resulting in large reductions of the native riparian plant communities [35]. From the northern portion of the CRD to the confluence with the Rio-Hardy, vegetation is predominantly salt cedar (*Tamarisk ramosissima*), arrowweed (*Pluchea sericea*), willow (*Salix gooddingii*) and cottonwood (*Populus fremontii*), with salt cedar, common reed (*Phragmites australis*) and cattail (*Typha domingensis*) predominating south of the confluence [36]. From the confluence with the Rio-Hardy to the Sea of Cortez, the Colorado River is still a perennial water flow. We divided the riparian zone into seven river segments (referred to here as reaches) based on geomorphic characteristics (see [37] for descriptions of each reach). Because the CRD is characterized by narrow and wide areas, dividing the corridor into reaches allowed us to separate potential scale-dependent effects and potential pixel shifts between satellite platforms, which were expected to more strongly influence narrower portions of the study area.

### 2.2. Mask Development and Data Acquisition

Five mask layers (regions) were digitized for the extraction of satellite EVI data. A mask of the CRD was digitized using a combination of the Basemap feature in ArcGIS v10.3 (ESRI, Inc., Redlands, CA, USA) and Google Earth (Google, Inc.; see [16] for a full description of this process). The mask excluded agricultural fields and heavily-disturbed/cleared areas, while retaining the remaining portions of the riparian zone and plant communities for each river reach (Figure 1). To isolate the response by land cover type, we created a mask of bare soil (24,435 ha) and agricultural fields (41,766 ha) and dissolved the riparian zone mask into a single vector feature representing riparian vegetation (30,694 ha). The final mask was a polygon digitized in ArcGIS v10.3 that included the entire MODIS and corresponding Landsat study region (Figure 1). All mask vector files were converted to 250-m rasters to match MODIS resolution prior to data extraction.

Landsat 5 TM and Landsat 8 OLI surface reflectance and surface reflectance-based EVI products (images) were acquired from the EROS Center Science Processing Architecture on Demand Interface (http://espa.cr.usgs.gov, accessed 28 February 2017). We selected two scenes from Path/Row 38/38 and 38/37 corresponding to the early (March–May), mid (June–August) and late (September–October) growing season for 2001–2016 (3 scenes per year), excluding 2012 (period between Landsat 5 decommissioning and the launch of Landsat 8; see Table 1 for acquisition periods used). We visually assessed all Landsat images and used only cloud-free scenes; therefore, the months chosen differed among some years.

**Figure 2:**
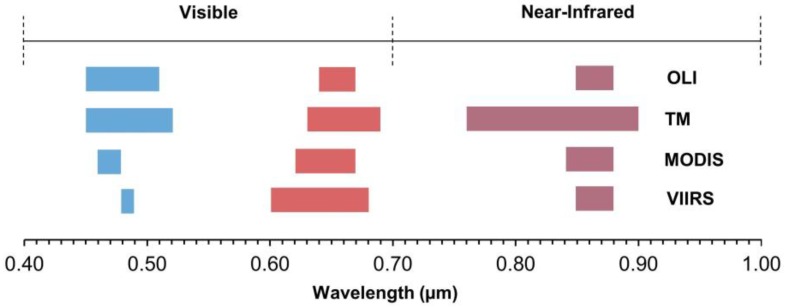
Visible (blue and red) and near-infrared band positions for Landsat 8 OLI, Landsat 5 TM, MODIS and VIIRS.

**Figure 3:**
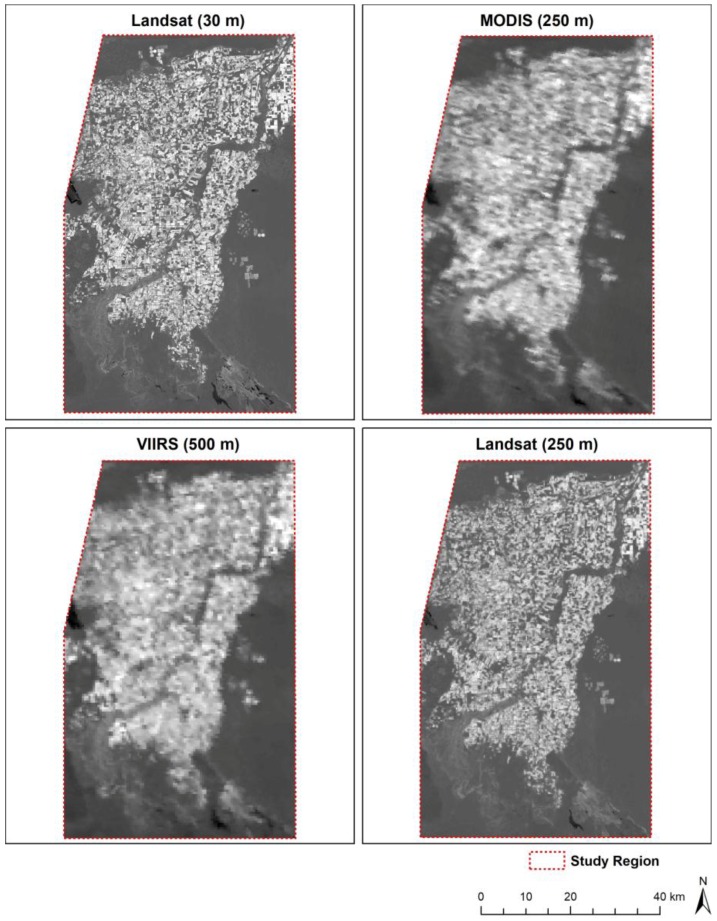
Example of EVI images acquired 22 April 2013 showing the differences in resolution from Landsat (native 30 m and upscaled 250 m), MODIS and VIIRS. VIIRS 500-m EVI is shown, as the native resolution is scaled to this resolution in the standard product.

Daily Terra MODIS surface reflectance data (MOD09 product) corresponding to the study area (tile h08v05) and the Landsat acquisition periods were acquired from the USGS Land Processes Data Active Archive Center (LP-DAAC). We used MOD09 to ensure correspondence in acquisition periods between the sensors (as opposed to the 16-day composited EVI product). Some MODIS scenes corresponding to the Landsat acquisition periods had excessive cloud cover. In these instances, we chose the highest quality scene occurring the day prior to or after the Landsat acquisition date (Table 1). We obtained MODIS daily surface reflectance NIR (Band 2; 250 m), red (Band 1; 250 m), blue (Band 3; 500 m), Quality Assessment (QA) and viewing geometry data, including sensor zenith angle (Vz) for each corresponding Landsat period (Table 1). VIIRS red and NIR bands (500 m) and the 1000-m blue band were obtained from the Land-Science Investigator-led Processing System (L-SISPS), as public data were not yet distributed regularly by the LP-DAAC. Although the VIIRS red and NIR bands are collected at 375 m and 750 m for the blue band, these data were upscaled to 500 m and 1000 m, respectively, by the L-SISPS. See Figure 2 for a comparison of bands between the four sensors and Figure 3 for a comparison of the resolution between Landsat, MODIS and VIIRS.

### 2.3. Data Pre-Processing

#### 2.3.1. MODIS and VIIRS EVI

All EVI data were generated using the standard MODIS Vegetation Index algorithm adapted to daily surface reflectance at the MODIS and VIIRS Principal Investigator Science Computing Facility (https://vip.arizona.edu). While this approach uses daily data with no compositing, the data filtering and QA approaches used in the standard MODIS VI algorithms were applied [4,18,27]. We downscaled the 500-m blue band (Band 3) to 250 m to match the resolution of Bands 1 and 2. VIIRS EVI data were downscaled in a similar fashion (250 m) using the red and NIR imagery (I1 and I2) and the blue band. All rasters were downscaled using bilinear interpolation. Although we also had clear, cloud-free Landsat images for most of the corresponding MODIS time periods, cloud or atmospheric conditions could have changed between Landsat and MODIS acquisition times; therefore, the QA information was used on each respective MODIS and VIIRS scene to remove clouds and areas with high aerosol content that are difficult to correct [38]. The retained pixels were cloud-free and only included observations with either low or average aerosol loads. This strict filtering approach aimed to standardize the datasets and promote accurate comparisons that minimized or eliminated noise-related differences. EVI was subsequently calculated for each scene using Equation 1. Because we were only interested in the vegetation signal, EVI values <0.0 (typically representing water or poorly-corrected data) were removed from the analysis, resulting in values ranging from 0–1.0.

#### 2.3.2. Landsat EVI

All Landsat EVI data processing techniques were performed in ArcGIS v10.3. The selected Landsat TM and OLI EVI scenes from Rows 37 and 38 were first combined into single rasters. The valid range of values of these scenes was from −1.0–+1.0. As with the MODIS data, we removed all negative values and any values above 1.0 (outside the valid range). To match the resolution of the MODIS EVI data, the Landsat rasters were upscaled from stock 30-m resolution to 250 m by taking the average of all cells falling within 250-m non-overlapping kernels. Although we visually selected clear, cloud-free Landsat scenes, we applied the MODIS QA mask described in Section 2.3 to remove pixels from the Landsat imagery corresponding to masked areas in the MODIS scenes.

### 2.4. Data Processing and Analysis

Mean MODIS, Landsat and VIIRS EVI data were extracted in ArcGIS using the masks (except land cover) described in Section 2.2. All scenes were registered to the same grid prior to data extraction. The result was the mean EVI of the masked areas for each MODIS, Landsat and VIIRS image. MODIS and Landsat EVI data from agricultural fields, bare soil and riparian vegetation were extracted and summarized using a mask and a simple data extraction program developed in-house at the University of Arizona’s Vegetation Index and Phenology Lab. The program scanned all pixels in the image, collected the ones that corresponded to the mask and then performed the necessary statistical analysis.

We used linear regression in SigmaPlot v13.0 (Systat Software, Inc., San Jose, CA, USA) to compare EVI and individual bands by sensor for the project area, each river reach and for the three dominant land cover types present: bare soil, agricultural fields and riparian vegetation (Landsat and MODIS). We also compared EVI estimates for the early, mid and late growing periods to assess seasonal variation (Table 1). Goodness-of-fit was reported as the coefficient of determination (R^2^), Percent Mean Relative Error (PMRE), bias and *p*-value for the regression coefficient (slope) of each equation. Bias and PMRE were calculated as:

(2) $$\mathrm{Bias}=\frac{1}{n}\sum_{i=1}^{n}\left(S1_{i}-S2_{i}\right),$$

(3) $$\mathrm{PMRE}=\frac{100}{n}\sum_{i=1}^{n}\left|\frac{S1_{i}-S2_{i}}{S2_{i}}\right|,$$

where *S*1 is EVI or band reflectance estimated by Landsat or VIIRS and *S*2 is from MODIS.

To address band-specific differences between MODIS and Landsat, we calculated the Simple Ratio (NIR/red) and plotted the NIR and red band reflectance values for each land cover type. Due to the large sensor swath and bowtie effect that result in large footprints at the edge of the sensor scan [39], we compared sensor Vz to PMRE, with the assumption that error would increase as Vz increased.

## 3. Results

### 3.1. Comparison by Study Region

**Figure 4:**
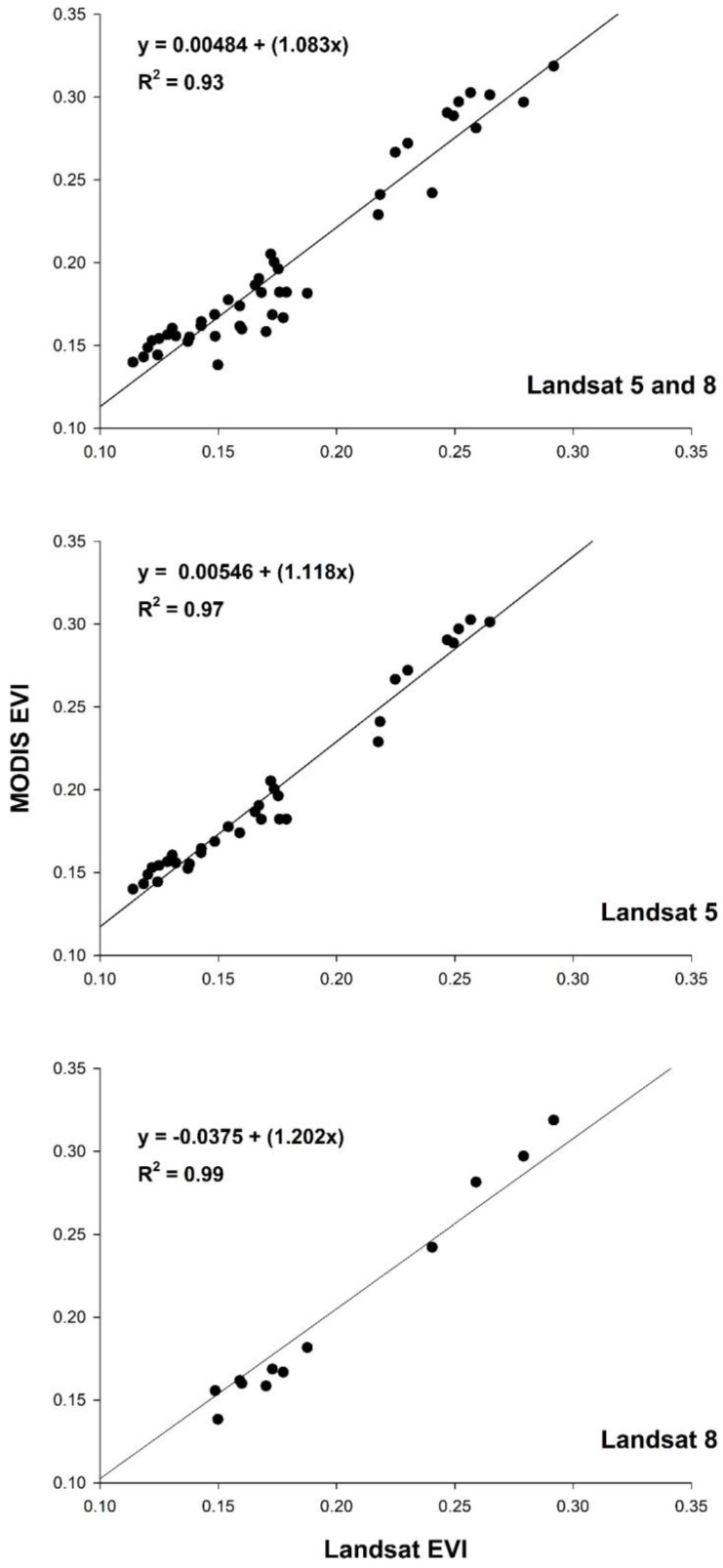
MODIS vs. Landsat 5 and 8 EVI (2000–2016) for the study region. Lines of the best fit are shown.

The correlation between MODIS and Landsat EVI for all years within the study region was high, but displayed some variability, with an R^2^ of 0.93 (*p* < 0.05) and a PMRE of 10.89% (Figure 4). When separating the response by sensor, this relationship improved to an R^2^ of 0.97 for Landsat 5 (2001–2011; Figure 4) and 0.99 for Landsat 8 (2013–2016; Figure 4), with a PMRE of 13.12% and 4.77% compared to MODIS, respectively. Bias for Landsat 5 was −0.0258 and −0.0028 for Landsat 8. There was no relationship between Vz and PMRE across all dates (mean Vz = 56.30°; R^2^ = 0.02; *p* > 0.05).

**Figure 5:**
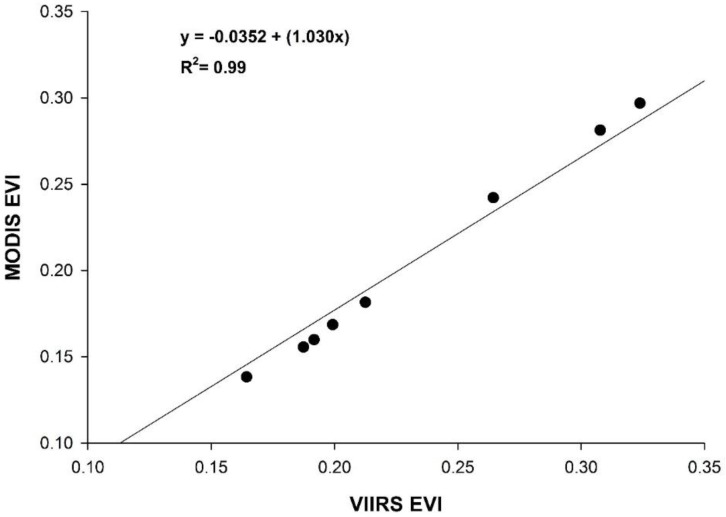
MODIS and VIIRS EVI of the study region from 2012–2016. The line of the best fit is shown.

The correlation between MODIS and VIIRS-based EVI was high (R^2^ = 0.99; *p* < 0.05; Figure 5), with a PMRE of 15.27%. VIIRS EVI estimates were consistently higher than MODIS, with an average bias of +0.0284. VIIRS errors were lowest during the early growing season, increasing through the mid and late portions of the growing period (see Table 2 for PMRE and bias by season).

We also observed different relationships between MODIS and Landsat for the early, mid and late growing seasons, with R^2^ values of 0.87, 0.21 and 0.21, respectively. The seasonal relationship for Landsat 5 was higher, with R^2^ values of 0.94, 0.74 and 0.53 for the early, mid and late growing season, respectively. This compared to 0.96, 0.88 and 0.48 for Landsat 8. See Table 2 for PMRE and bias by sensor and season.

**Figure 6:**
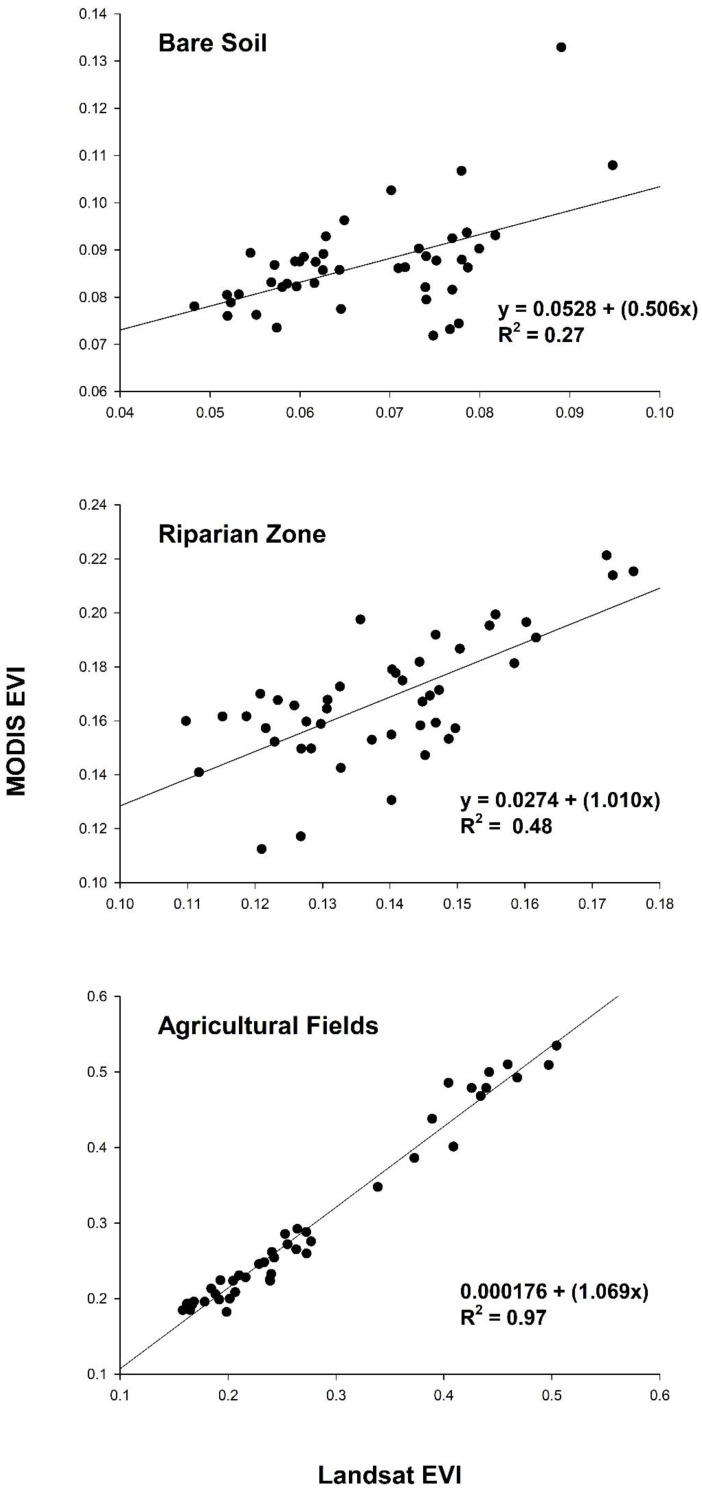
Landsat vs. MODIS EVI for the three predominant land cover types in the study region. Lines of the best fit are shown.

**Figure 7:**
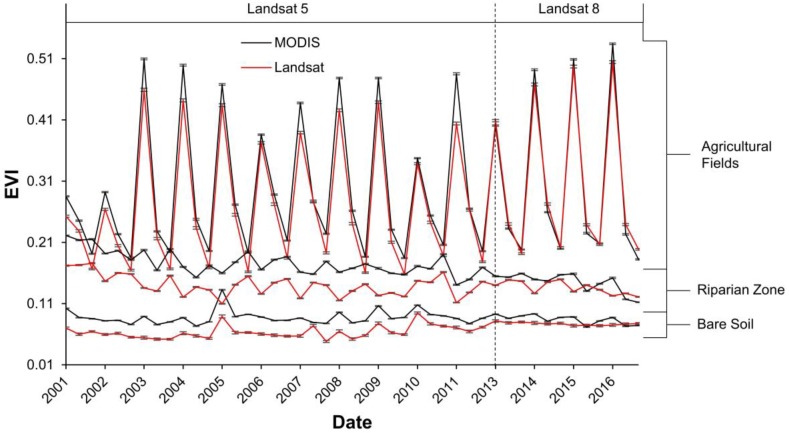
Time series of MODIS and Landsat EVI for the growing period of 2001–2016. Bars show the standard error.

**Figure 8:**
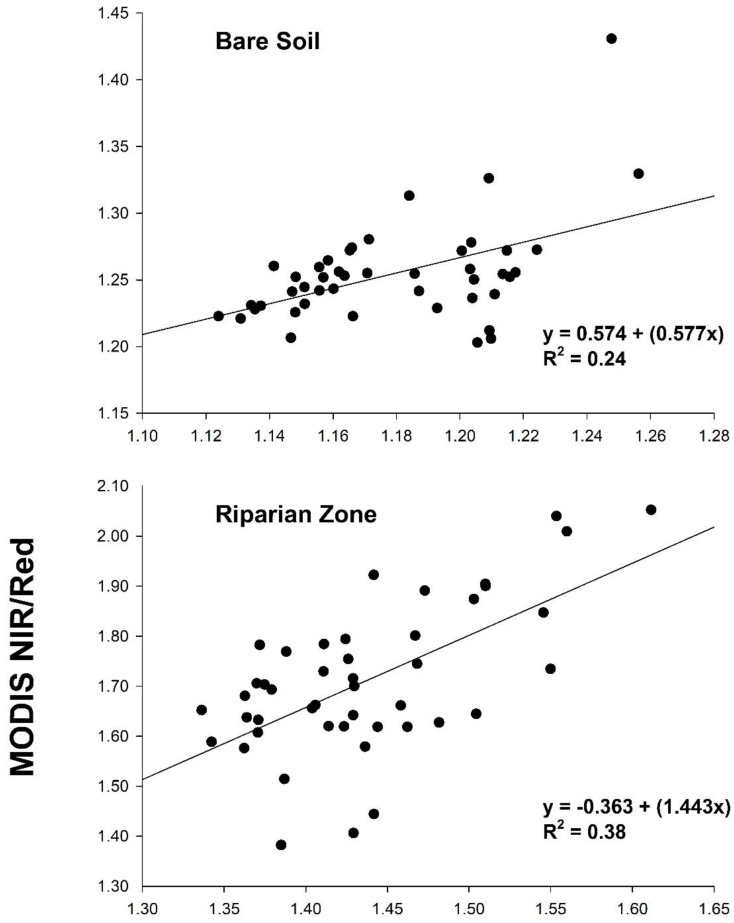

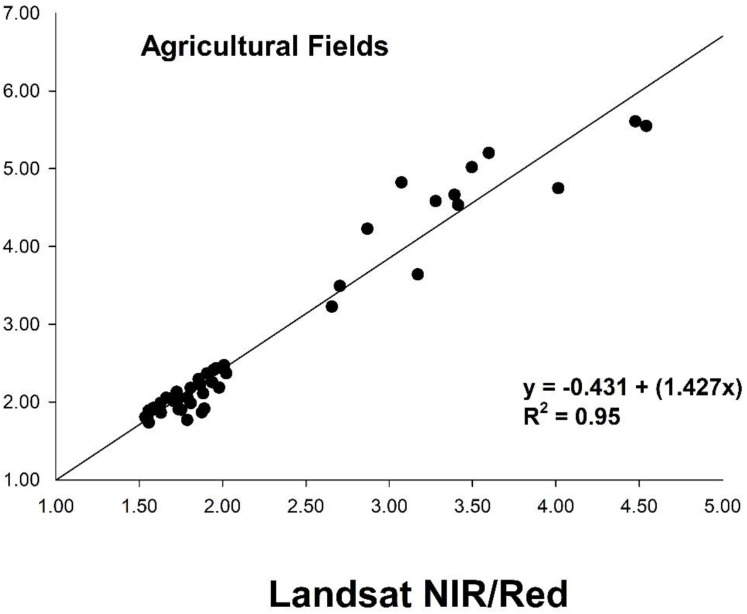
Landsat vs. MODIS simple ratio (NIR band/red band) for the three predominant land cover types in the study region. Lines of the best fit are shown.

**Figure 9:**
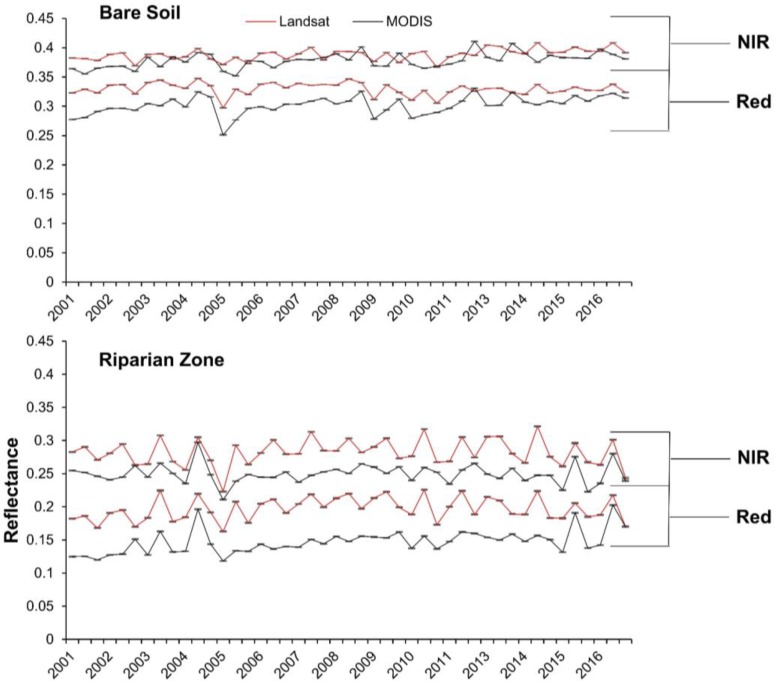

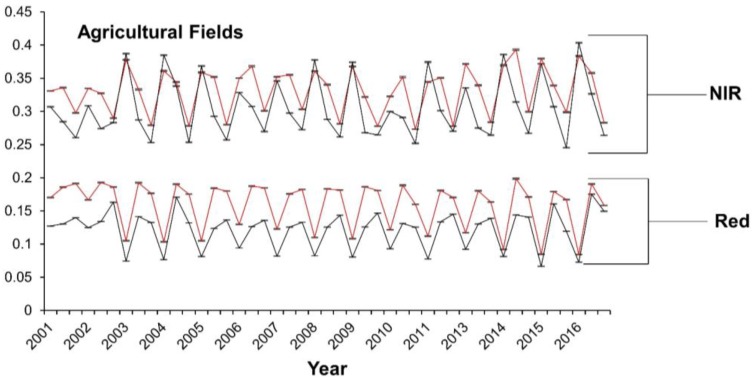
Time series of MODIS and Landsat NIR and red bands for the three predominant land cover types surveyed. Bars are the standard error.

Landsat EVI was highly correlated with MODIS for agricultural fields (R^2^ = 0.97; *p* < 0.001), while the relationship was weaker for bare soil (R^2^ = 0.27; *p* < 0.001) and riparian vegetation (R^2^ = 0.48; *p* < 0.001; Figure 6 and Figure 7). Annual trends between MODIS and Landsat 5 EVI for riparian vegetation and bare soil were relatively synchronous, but less so for Landsat 8 (Figure 7). For the simple ratio (NIR/red), bare soil had the weakest correlation and agricultural fields had the strongest (Figure 8). The MODIS and Landsat blue, red and NIR bands were moderately correlated (R^2^ = 0.47, 0.57 and 0.40, *p* < 0.001, respectively). The red band deviated more than the NIR in each land cover type, with both red and NIR displaying a positive bias (Table 3; Figure 9).

### 3.2. Comparisons by River Reach

Relationships in EVI between MODIS and Landsat were more variable and had lower correlations by reach than for the study region (Table 4). For Landsat 5 and 8 combined, Reach 1 had the lowest correlation (R^2^ = 0.01), while Reach 7 had the highest (R^2^ = 0.74). Except for Reach 4, the correlation between Landsat 5 and MODIS was higher than for Landsat 8 (see Table 4 for specific values); however, Landsat 8 had a lower PMRE across reaches (Table 4).

Correlations between MODIS and VIIRS EVI were higher by reach than for Landsat, with the highest in Reach 4 (R^2^ = 0.98) and the lowest in Reach 1 (R^2^ = 0.89). PMRE ranged from 14.19% in Reach 2 to 22.61% in Reach 7 (Table 4). VIIRS EVI estimates were higher than MODIS in every reach.

## 4. Discussion

### 4.1. Comparison by Study Region

Correlations between MODIS and Landsat EVI were high, but further improved when separated by sensor, including a near perfect relationship with Landsat 8 (Figure 4). Similarly, the PMRE in EVI also differed, with Landsat 5 substantially higher than Landsat 8. This finding is consistent with differences we expected based on the band designations and radiometric resolution of the three platforms (Figure 2). While the red band is similar between the three sensors, MODIS’ NIR band is closer in range to Landsat 8 than to Landsat 5 (0.84–0.88, 0.85–0.88 and 0.76–0.90 μm, respectively; Figure 2; [19,40]). Both MODIS and Landsat 8 OLI are 12-bit sensors, compared to 8-bit quantization for Landsat 5 TM. Though these spectral and radiometric characteristics help explain the greater agreement between Landsat 8 and MODIS, it is worth noting fewer OLI scenes (*n* = 12) were analyzed compared to Landsat 5 (*n* = 33).

Correlations differed by growing season for Landsat, with the early part having the highest correlation with MODIS EVI. The mid and late growing seasons coincide with the onset of the monsoon, which is marked by a significant increase in atmospheric moisture and a potential increase in Aerosol Optical Thickness (AOT), including sub-pixel clouds that are not removed by the QA filtering due to non-detection by the cloud mask algorithm [41]. This likely led to higher correspondence during the early season and lower correspondence during the later period.

Atmospheric correction (surface reflectance) techniques applied to these sensors could also explain observed differences [38]. Though surface reflectance correction for both Landsat 5 and 8 is based on the Second Simulation of a Satellite Signal in the Solar Spectrum radiative transfer model, OLI data are further refined using an internal algorithm that takes advantage of the platform’s additional, narrower bands and improved radiometric and signal-to-noise ratio [42]. Landsat 8 OLI Surface Reflectance Code (LaSRC) differs from the code applied to Landsat 5 TM (i.e., Landsat Ecosystem Disturbance Adaptive Processing System (LEDAPS); [43]) in a few key ways: first, LEDAPS corrects for water vapor and air temperature using the National Centers for Environmental Prediction grid, while LaSRC uses the MODIS Climate Modelling Grid (CMG) for both of these parameters. To correct for AOT, LEDAPS incorporates the correlation between chlorophyll absorption and bound water absorption of the scene, while LaSRC employs the MODIS CMG, resulting in greater agreement between OLI and MODIS surface reflectance data [42].

Although Vz was not correlated with PMRE, this could be due to a lack of variation in view angles. Five scenes ranged from a scene average of 6.17–7.98°, while the remaining 40 scenes ranged from 62.02–62.80°. The impact of the sensor Vz remains indirect, in that it makes the observations used in this analysis larger, suggesting more adjacency impact than the nominal 250-m grid pixels. This issue complicates the correlation with near-nadir sensors like TM and OLI. 

Because VIs are designed to respond to vegetation, it is not surprising we found lower correlations between MODIS and Landsat EVI over bare soil and sparsely-vegetated areas like the riparian zone (Figure 6) [44], where the soil color is the dominant driver of the red and NIR spectral response [9]. Based on NIR/red reflectance, soil likely contributed to increased variability and decreased correspondence between platforms, as correlations for NIR/red and EVI systemically increased with vegetation density (Figure 6 and Figure 8) [9,10,44]. Despite a stronger correlation between surface reflectance in the red wavelengths, the red bands displayed greater error rates compared to the NIR (Table 3). This is contrasted by [21], who found higher error with MODIS’ NIR band when compared to field-based measurements.

### 4.2. Comparisons by River Reach

Compared to the study region, comparisons by reach showed lower agreement between platforms (Table 4). In the case of Landsat and MODIS, the correlation was lower in smaller, narrower reaches (e.g., 1–4) compared to the larger, wider ones (e.g., 5–7). This can likely be attributed to a combination of factors. First, pixel geolocation accuracy (registration) is expected to have the greatest effect in narrow areas considering MODIS averages about a 50-m geolocation error at 250 m [39]. Reaches 1, 3 and 4 are one cell wide in the narrowest portions, making pixel misalignment particularly pervasive in these areas. Pixel-level comparisons (not shown) were also conducted to provide insight into such differences, but co-registration error was too great to provide meaningful results. Second, the upscaling of cells from 30–250 m could be influenced by the level of landscape heterogeneity. Because the riparian zone within the CRD is lined extensively by agricultural fields (see Figure 1), this likely affected most, if not all reaches, with a greater effect on the narrow reaches. Pixel alignment and scaling likely also help explain the differences in correlations observed for the three different land cover types and the lower correlations observed for bare soil and riparian vegetation (sampling regions for these two land cover types were narrower than for agricultural areas). Riparian vegetation in the study region was also more heterogeneous in spatial distribution, likely decreasing the correspondence between platforms. Band position and width also explain these differences, especially over non- to sparsely-vegetated pixels, where the spectral response is at its lowest.

Unlike Landsat, VIIRS displayed a high correlation with MODIS EVI throughout Reaches 1–7 (Table 4). Similar resolution and ±200-m geolocation error between sensors likely reduced pixel scaling effects and misalignment between datasets. VIIRS consistently showed higher EVI compared to MODIS for these vegetated areas, which is consistent with the preliminary evaluation of this sensor [45,46]. As expected, VIIRS errors in Reaches 1–4 were lower than those for Landsat 5 and 8 (Table 4); however, VIIRS displayed higher error than Landsat in Reaches 5–7 (the largest reaches). The comparatively lower errors by Landsat are likely due to the reduced effect of pixel misalignment and upscaling over larger areas (discussed above), while the larger PMRE by VIIRS could be due to real differences in the landscape as observed by these comparable sensors. This is also consistent with our findings for the study region. In the future, significance testing between mean estimates of EVI (and reflectance by band) for each sensor would help assess these differences.

## 5. Conclusions

Despite variability due to differences in factors such as geolocational accuracy, spatial resolution and spectral characteristics, Landsat- and VIIRS-based EVI data were highly similar to MODIS EVI over large areas and verdant, homogenous vegetation. This strong relationship degraded over smaller, sparsely-vegetated areas for Landsat. Therefore, caution should be exercised when using higher resolution Landsat EVI data over heterogeneous areas with low vegetation densities, such as those commonly encountered in arid and semi-arid environments. When compared to MODIS, both Landsat and VIIRS displayed varying degrees of error, which could affect biophysical analyses of vegetation (e.g., quantification of evapotranspiration or biomass), and this will likely be exacerbated in heterogeneous, smaller areas. Our work suggests that continuity and translation across these sensors and indices are possible and practical, as they are linear with small to moderate biases. With the aforementioned limitations in mind, Landsat and VIIRS perform similarly to MODIS when used for landscape-scale estimation of EVI. This is a topic of particular interest as our observational time series continues to grow across different sensors.

## Figures and Tables

**Table 1:** Acquisition periods of all MODIS, Landsat and VIIRS scenes analyzed (2001–2016). Year 2012 was not analyzed due to the transition from Landsat 5 to Landsat 8. * Acquisition period(s) differed between platforms. ** Image not analyzed due to insufficient quality.

Year	Sensor(s)	Acquisition Date(s)
2001	MODIS, Landsat 5 TM	7 May, 26 July, 12 September
2002 *	MODIS, Landsat 5 TM	10 May, 29 July
	MODIS	14 September
	Landsat	15 September
2003	MODIS, Landsat 5 TM	26 March, 16 July, 4 October
2004 *	MODIS, Landsat 5 TM	12 March, 6 October
	MODIS	1 July
	Landsat 5 TM	2 July
2005	MODIS, Landsat 5 TM	15 March, 5 July, 9 October
2006	MODIS, Landsat 5 TM	19 April, 8 July, 26 September
2007	MODIS, Landsat 5 TM	6 April, 11 July, 13 September
2008	MODIS, Landsat 5 TM	23 March, 29 July, 17 October
2009	MODIS, Landsat 5 TM	26 March, 1 August, 20 October
2010	MODIS, Landsat 5 TM	30 April, 3 July, 7 October
2011	MODIS, Landsat 5 TM	16 March, 22 July, 26 October
2013	MODIS, Landsat 8 OLI, VIIRS	22 April, 12 August, 31 October
2014	MODIS, Landsat 8 OLI, VIIRS	8 March, 28 Jun, 2 October
2015 *	MODIS, Landsat 8 OLI, VIIRS	27 March
	MODIS, Landsat 8 OLI	19 September
	MODIS	16 July
	Landsat 8 OLI	17 July
2016 *	MODIS, Landsat 8 OLI	13 March
	VIIRS	11 March **, 23 October
	MODIS	18 July, 22 October
	Landsat 8 OLI	19 July, 23 October

**Table 2:** Percent Mean Relative Error (PMRE; %) and bias in EVI values of the study region between Landsat, VIIRS and MODIS for the early, mid and late portions of the growing season. The number of scenes analyzed for each period is given by *n*.

	Landsat 5			Landsat 8			VIIRS		
Season	PMRE	Bias	*n*	PMRE	Bias	*n*	PMRE	Bias	*n*
**Early**	13.29	−0.0353	11	5.76	−0.0172	4	9.23	+0.0252	3
**Mid**	9.62	−0.0171	11	4.94	+0.0082	4	17.61	+0.0308	2
**Late**	16.46	−0.0249	11	3.60	+0.0005	4	19.74	+0.0299	3

**Table 3:** Percent Mean Relative Error (PMRE; %) and bias (reflectance) between MODIS and Landsat for the red and NIR bands for the three predominant land cover types in our study region.

Land Cover Type	PMRE (Red Band)	Bias (Red Band)	PMRE (NIR Band)	Bias (NIR Band)
**Bare Soil**	9.49	+0.03	3.78	+0.01
**Riparian Vegetation**	34.84	+0.05	13.27	+0.03
**Agricultural Fields**	31.80	+0.04	10.63	+0.03

**Table 4:** R^2^, Percent Mean Relative Error (PMRE; %) and bias between MODIS and Landsat 5, Landsat 8 and VIIRS EVI by each river reach. Thirty-three Landsat 5, 12 Landsat 8 and 8 VIIRS scenes were analyzed. * Significant at the 0.05 level. GFM is the Goodness-of-Fit Measure.

Sensor	GFM	Reach 1	Reach 2	Reach 3	Reach 4	Reach 5	Reach 6	Reach 7
**Landsat 5**	R^2^	0.02	0.27 *	0.49 *	0.02	0.86 *	0.83 *	0.86 *
	PMRE	25.00	29.89	29.45	20.97	18.31	15.96	17.77
	Bias	−0.0529	−0.0526	−0.0530	−0.0506	−0.0378	−0.0268	−0.0278
**Landsat 8**	R^2^	0.00	0.24	0.46 *	0.25	0.63 *	0.81 *	0.84 *
	PMRE	16.40	19.33	21.37	16.20	7.64	4.67	3.94
	Bias	−0.0359	−0.0331	−0.0354	−0.0324	−0.0055	+0.0039	+0.0023
**VIIRS**	R^2^	0.89 *	0.93 *	0.97 *	0.98 *	0.91 *	0.91 *	0.96 *
	PMRE	14.48	14.19	15.65	16.07	20.35	20.19	22.61
	Bias	+0.0244	+0.0223	+0.0235	+0.0321	+0.0347	+0.0284	+0.0268
